# Brownian Motion in a Speckle Light Field: Tunable Anomalous Diffusion and Selective Optical Manipulation

**DOI:** 10.1038/srep03936

**Published:** 2014-02-05

**Authors:** Giorgio Volpe, Giovanni Volpe, Sylvain Gigan

**Affiliations:** 1 Institut Langevin, UMR7587 of CNRS and ESPCI ParisTech, 1 rue Jussieu, 75005 Paris, France; 2 Physics Department, Bilkent University, Çankaya, 06800 Ankara, Turkey

## Abstract

The motion of particles in random potentials occurs in several natural phenomena ranging from the mobility of organelles within a biological cell to the diffusion of stars within a galaxy. A Brownian particle moving in the random optical potential associated to a *speckle pattern*, i.e., a complex interference pattern generated by the scattering of coherent light by a random medium, provides an ideal model system to study such phenomena. Here, we derive a theory for the motion of a Brownian particle in a speckle field and, in particular, we identify its universal characteristic timescale. Based on this theoretical insight, we show how speckle light fields can be used to control the anomalous diffusion of a Brownian particle and to perform some basic optical manipulation tasks such as guiding and sorting. Our results might broaden the perspectives of optical manipulation for real-life applications.

Various phenomena rely on particles performing stochastic motion in random potentials. Examples range from the nanoscopic world of molecules undergoing anomalous diffusion within the cytoplasm of a cell^1^ to the Brownian motion of stars within galaxies^2^. Another example of this kind of phenomena is given by the motion of a Brownian particle in a random optical potential generated by a speckle pattern, i.e., the random light field resulting from complex light scattering in optically complex media, such as biological tissues, turbid liquids and rough surfaces (see background in Figure 1a–c)^3^,^4^. This latter example is particularly suited to work as a model system because its parameters (e.g., particle size and material, illumination light) are easily controllable and its dynamics are easily accessible by standard optical microscopy techniques^5^. Earlier experimental works showed the possibility of trapping particles in high-intensity speckle light fields^6^,^7^,^8^,^9^, the simplest optical manipulation task, and the emergence of superdiffusion in an active media constituted by a dense solution of microparticles that generates a time-varying speckle field^10^. However, apart from these previous studies, there is little understanding of the interaction of Brownian motion with random light potentials and the intrinsic randomness of speckle patterns is largely considered a nuisance to be minimized for most purposes, e.g., in optical manipulation^11^,^12^. In fact, similar and even more complex effects have been extensively studied using periodic potentials rather than random potentials: these studies include the observation of giant diffusion induced by an oscillating periodic potential^13^, and the demonstration of guiding and sorting particles using either moving periodic potentials^14^,^15^,^16^ or static periodic potentials in microfluidic flows^17^,^18^,^19^. It is not a priori obvious that the same phenomena that have been observed with periodic potentials can also arise with random potentials, as the statistical properties of random potentials fundamentally differ from those of periodic potentials. For example, in non-equilibrium statistical physics, the dynamics of a Brownian particle in a moving periodic potential can be described as a straightforward generalization of the dynamics of a Brownian particle in static not out-of-equilibrium potentials, while this is no longer the case for random potentials for which a full out-of-equilibrium description is still required^20^.

In this Letter, we study the Brownian motion of a particle in a speckle pattern and, in particular, we derive the characteristic timescale τ of such motion, which is universal as it depends only on the universal properties of speckle light fields^3^,^21^,^22^. This theoretical insight permits us to demonstrate that random potentials can also be used instead of more regular potentials to control the dynamics of a Brownian particle in a very accurate way, despite their intrinsic randomness. First of all, while the emergence of superdiffusion in time-varying speckle patterns has already been observed^10^, we demonstrate that speckle fields provide a tunable and controllable model system to study anomalous diffusion by relating τ to the emergence of subdiffusion and superdiffusion in time-varying speckle patterns. Moreover, we demonstrate the possibility of harnessing the *memory effect* of speckle fields^21^,^22^ to perform basic optical manipulation tasks such as guiding and sorting^23^, which go beyond selective optical trapping in high-intensity speckle grains^7^,^8^.

## Results

Speckle light patterns can be generated by different processes, such as scattering of a laser on a rough surface, multiple scattering in an optically complex medium, or mode-mixing in a multimode fiber^4^. In general, they are the result of the interference of a large number of waves propagating along different directions and with a random phase distribution, and, despite their random appearance, they share some universal statistical properties^3^,^24^ (see Supplementary Note S1). In particular, a speckle pattern has a negative exponential intensity distribution, and the normalized spatial autocorrelation function *C_I_*(Δ**r**) can be approximated by a Gaussian^25^: 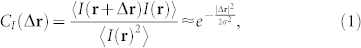
where *I*(**r**) is the speckle pattern intensity as a function of the position **r** and the standard deviation *σ* ≈ *d*/3 is proportional to the average speckle grain size *d* (see Supplementary Note S1).

The motion of a Brownian particle in a static speckle field is the result of random thermal forces and deterministic optical forces**.** In the following discussion, we focus on particles whose radius is smaller than the light wavelength λ. We develop a theory that is strictly valid for Rayleigh particles (see Methods), but our conclusions hold also for larger particles^26^. In particular, as we show in the Supplementary Figure S1 using exact electromagnetic theory^27^,^28^, our theory is also approximately valid for bigger particles provided that their radius is smaller than the light wavelength λ. Optical gradient forces are the dominant deterministic forces acting on small particles, and they attract high-refractive index particles towards the intensity maxima of the optical field^26^.

**Figure 1:**
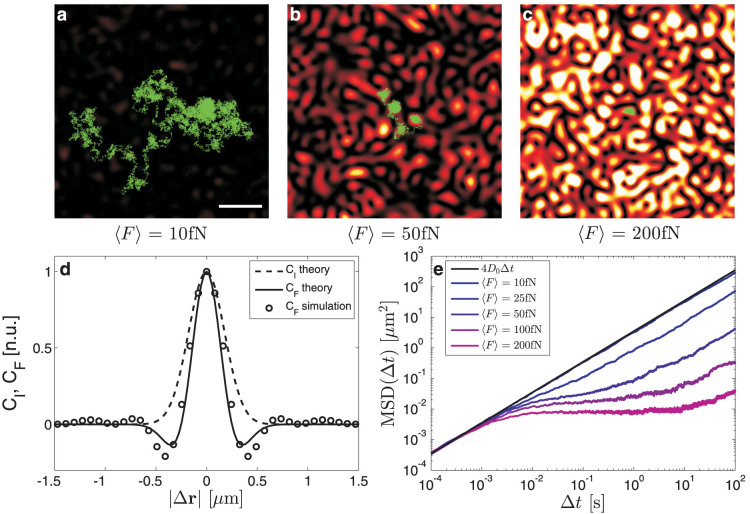
Subdiffusion in a static speckle pattern. (a–c) The background represents a speckle pattern generated by a circular aperture (*λ* = 1064 nm, speckle grain 490 nm); the white scale bar corresponds to 2 μm. The trajectories (green solid lines) show progressive confinement of a polystyrene bead (R = 250 nm, *n_p_* = 1.59) in water (*n_m_* = 1.33, *η* = 0.001 Ns/m^2^, T = 300 K) as a function of the increasing speckle intensity corresponding to an average force on the particle of (a) 〈F〉 = 10 fN (〈I〉 = 13 mW/μm^2^), (b) 〈F〉 = 50 fN (〈I〉 = 65 mW/μm^2^), (c) 〈F〉 = 200 fN (〈I〉 = 260 mW/μm^2^). (d) Normalized autocorrelation function of the force field produced by the speckle pattern according to our theoretical model (solid line) and in the simulated speckle pattern (circles). The dashed line represents the theoretical normalized autocorrelation function of the speckle pattern intensity. (e) Brownian particle mean square displacements as a function of 〈F〉 (purple lines), and their deviation from Einstein's free diffusion law (black line).

As a particle moves in a speckle field, the optical force acting on it changes both in magnitude and direction with a characteristic time scale 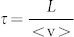, where *L* is the correlation length of the optical force field and <v> is the average particle drift speed. The optical force field correlation function (Figure 1d) is 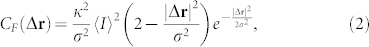
where 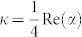 and *α* is the particle polarizability, so that 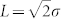 (see detailed derivation in Methods and Supplementary Note S2). Since the particle motion is overdamped^29^, the average particle drift speed is 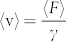, where *γ* is the particle friction coefficient and 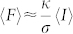 is the average force (see Supplementary Note S2). Thus, we obtain 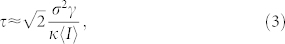
which is our central theoretical result and permits us to demonstrate how random optical fields can also be used instead of more regular ones to control the dynamics of a Brownian particle, despite the intrinsic randomness of the field. In the following, after considering how *τ* is related to the Brownian motion of a particle in a static speckle pattern, we will present two such examples via numerical experiments.

We start by considering the motion of a particle in a static speckle pattern. As shown by the trajectory in Figure 1a, when the optical forces are relatively low (average force 〈*F*〉 = 10 fN), the particle is virtually freely diffusing. As the forces increase (Figure 1b, 〈*F*〉 = 50 fN), first a subdiffusive behavior emerges where the particle is metastably trapped in the speckle grains, while it can still move between them^30^. Finally, for even stronger forces (Figure 1c, 〈*F*〉 = 200 fN), the particle remains trapped in one of the speckle grains for a very long time, as previously observed experimentally^6^,^7^,^8^,^9^. These observations can be interpreted in terms of *τ*: for relatively high forces (〈*F*〉 = 200 fN, *τ* ≈ 5.6 ms), *τ* is quite low which means that the particle, on average, experiences a restoring force towards its previous position quite soon in its motion, having little possibility to escape a speckle grain; for relatively low forces (〈*F*〉 = 10 fN, *τ* ≈ 112.5 ms) instead, *τ* is much higher which means that the particle has time to diffuse away from a speckle grain before actually experiencing the influence of the optical forces exerted by it. These qualitative considerations can be made more precise by calculating the mean square displacement MSD(Δ*t*) of the particle motion (see Methods). As shown in Figure 1e, for low optical forces and high *τ* the mean square displacement is substantially linear in Δt, i.e. MSD(Δ*t*) ≈ 4*D_SE_*Δ*t*, where *D_SE_* is the Stokes-Einstein diffusion coefficient. As the forces increase and *τ* decreases, there is a transition towards a subdiffusive regime characterized by MSD(Δ*t*) ∝ Δ*t^β^* with *β* < 1. We remark that, for very large Δ*t*, the motion returns diffusive, i.e. *β* = 1, albeit with an effective diffusion coefficient *D*_eff_ < *D_SE_*. Interestingly, in many naturally occurring anomalous diffusion processes, such as the subdiffusion of molecules within the living cell, the distribution of waiting times is expected to correspond to a random walker continually caught in potential wells whose depths are distributed exponentially^1^. Compared to periodic potentials, therefore, speckle patterns represent better model systems to study such phenomena: in fact, while periodic potentials are characterized by only a few potential depths, the distribution of potential depths (e.g. the distribution of intensities) in a speckle pattern follows a negative exponential distribution^3^,^24^.

**Figure 2:**
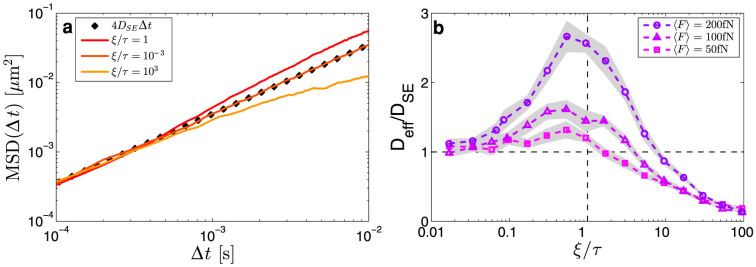
Superdiffusion in a time varying speckle pattern. (a) Mean square displacements in logarithmic scale for a Brownian particle moving in a speckle pattern which varies on a timescale *ξ* ≈ *τ* (red line), *ξ* ≪ *τ* (orange line), and *ξ* ≫ *τ* (yellow line). The dots represent Einstein's free diffusion law. (b) The effective diffusion of the motion at long timescales as a function of *ξ*/*τ* shows a transition from subdiffusion (*D*_eff_ < *D_SE_*) to superdiffusion (*D*_eff_ > *D_SE_*). The maximum value of the superdiffusion appears for *ξ* ≈ *τ* (*τ* ≈ 22.5 ms for 〈F〉 = 50 fN, *τ* ≈ 11.2 ms for 〈F〉 = 100 fN and *τ* ≈ 5.6 ms for 〈F〉 = 200 fN). Every mean point is averaged over 500 particle trajectories 100 s long, and whose initial position was randomly chosen within the speckle field. The gray shaded areas represent one standard deviation around the average values.

We move now to an example of fundamental interest: the demonstration that using speckle patterns it is possible to control and tune anomalous diffusion continuously from subdiffusion to superdiffusion by employing a *time-varying* speckle pattern which changes over a timescale *ξ* similar to *τ*. A time-varying speckle pattern can result from a time-varying environment^10^, but can also be produced in a more controllable way by modulating spatially or spectrally the laser that generates it^4^. Here, we consider timescales for the speckle pattern variation in the order of microseconds or even slower: this is several orders of magnitude above the fluctuation timescales observed for incoherent light, which result in a loss of contrast and of the corresponding optical forces^31^. As can be seen in Figure 2a, different diffusive regimes emerge depending on the value of the ratio *ξ*/*τ*, thus allowing one to tune the diffusive behavior of the particle just relying on the external control of the speckle pattern time scale *ξ*. For *ξ*/*τ* ≫ 1, the speckle pattern motion is adiabatic so that the particle can reach its equilibrium distribution in the optical potential before the speckle field changes. This leads to a subdiffusive behavior, i.e., MSD(Δ*t*) ∝ Δ*t^β^* with *β* < 1, as in a static speckle pattern. For *ξ*/*τ* ≪ 1, the particle cannot follow the fast variation of the speckle pattern so that the average optical force on the particle is zero leading to a diffusive behavior, i.e., MSD(Δ*t*) ≈ 4*D_SE_*Δ*t*. For *ξ*/*τ* ≈ 1, the particle is subject to time-varying forces, which can induce a superdiffusive behavior, i.e., MSD(Δ*t*) ∝ Δ*t^β^* with *β* > 1. As in the case of the static speckle field, at long timescales, the particle motion will become again diffusive. Figure 2b highlights the transition from subdiffusion to superdiffusion by plotting 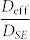 as a function of *ξ*/*τ* for various 〈*F*〉: if the resulting *D*_eff_ > *D_SE_*, the particle has undergone superdiffusion. In this way, the diffusive behavior of a Brownian particle in a speckle field can be controlled by tuning the relevant adimensional parameter *ξ*/*τ*, thus providing a simple model system to study anomalous diffusion^32^, which has been shown to occur naturally, e.g., in the kinetics of single molecules in living cells^1^.

**Figure 3:**
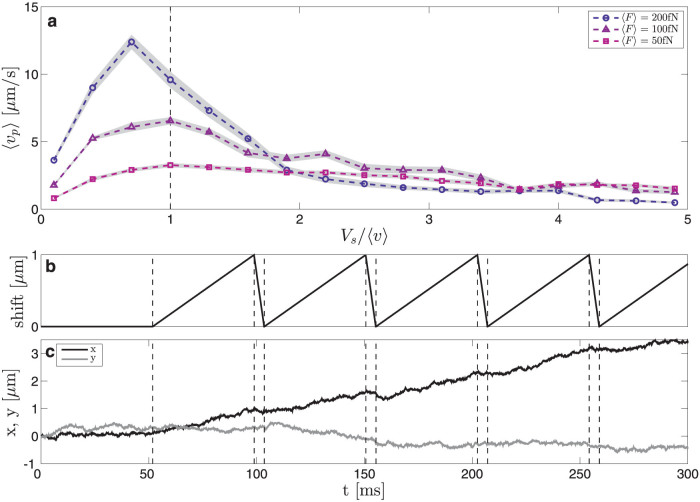
Guiding by the speckle memory effect. (a) Average guiding velocity 〈*v_p_*〉 in the direction of the speckle pattern shift as a function of the shift speed *V*_S_ for 〈F〉 = 200 fN (circles), 〈F〉 = 100 fN (triangles), and 〈F〉 = 50 fN (squares). The maximum 〈*v_p_*〉 is achieved for *V*_S_ ≈ 〈*v*〉 (〈*v*〉 ≈ 10.5 μm/s for 〈F〉 = 50 fN, 〈*v*〉 ≈ 21.2 μm/s for 〈F〉 = 100 fN and 〈*v*〉 ≈ 42.4 μm/s for 〈F〉 = 200 fN). Every mean velocity point is calculated over 500 trajectories simulated during 10 s and whose initial position was randomly chosen within the speckle field. The gray shaded areas represent one standard deviation around the average values. (b) Speckle pattern shift and (c) particle displacement as a function of time in the direction of the speckle pattern shift *x* (black line) and in the orthogonal direction *y* (gray line). The speckle pattern repeatedly shifts first slowly in the positive direction by 1 μm, and then fast to the initial position. The dashed lines represent the instant of time when there is a change of trend in the speckle pattern shift.

The second example is more applied and is the demonstration that it is possible to control the motion of a Brownian particle by using speckle fields, which sets the stage to perform optical manipulation tasks such as guiding particles in a particular direction, despite the randomness of the illumination. The relevant parameter for guiding is the ratio between the speckle pattern speed *V*_S_ and the average drift velocity of the particle in a static speckle pattern 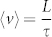. As *V*_S_/〈*v*〉 increases, the average guiding velocity 〈*v_p_*〉 reaches a maximum for *V*_S_/〈*v*〉 ≈ 1, as shown in Figure 3a. A small speckle pattern translation up to a few micrometers can be implemented capitalizing on the speckle property known as memory effect^21^,^22^: for a speckle pattern generated by a thin sample, a small tilt of the illumination, easily achievable, e.g., with a galvanometric mirror or an acousto-optic deflector, entails a small spatial translation of the speckle pattern. As shown in Figure 3b, this is sufficient to realize a Brownian ratchet^33^: the speckle pattern repeatedly shifts first slowly (*V*_S_/〈*v*〉 = 1) by 1 μm in the positive direction, which exerts a strong drag on the particle, and then fast (*V*_S_/〈*v*〉 = 10) back to the initial position, which has little effect on the particle position. In 250 ms, the particle is dragged by ≈3 μm in the direction of the speckle pattern shift, while the particle's trajectory in the perpendicular direction remains unaffected (Figure 3c).

**Figure 4:**
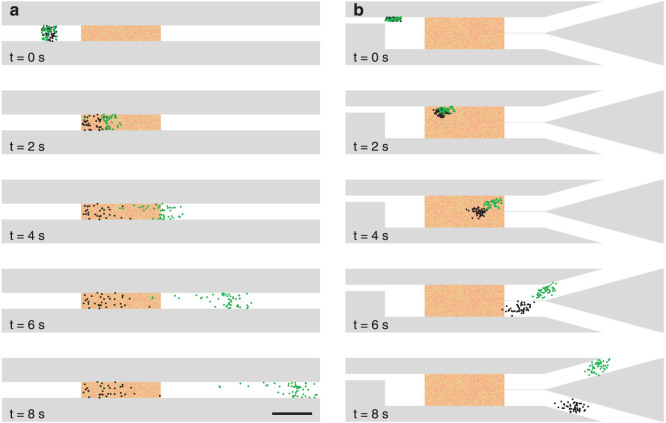
Microfluidic speckle sieve and speckle sorter. (a) Lapse-time snapshots of the motion of polystyrene particles with radius R = 200 nm (〈*F*〉 = 90 fN, green dots) and R = 250 nm (〈*F*〉 = 46 fN, black dots) in a microfluidic speckle sieve, where a static speckle pattern (red shaded area) traps the smaller particles while it lets the larger particles go away with the flow (flow speed 42 μm/s, see also Supplementary Video S1). (b) Same as in (a), but with the speckle pattern ratcheting in the direction orthogonal to the flow by 1 μm (flow speed 34 μm/s, see also Supplementary Video S2). The black scale bar represents 50 μm.

This guiding capability of a speckle pattern can be combined with standard microfluidic systems in order to perform tasks, such as sieving and sorting. For example, a static speckle pattern can be employed to realize a *speckle sieve* (Figure 4a and Supplementary Video S1). As a liquid containing 200 nm and 250 nm particles flows from left to right at 42 μm/s, a static speckle pattern efficiently holds the larger particles back while the smaller ones go through almost unaffected; interestingly, the size of the particle that are held back can be dynamically adjusted by changing the intensity of the speckle pattern. A spatiotemporal varying speckle field instead can be employed to realize a *speckle sorter* (Figure 4b and Supplementary Video S2). In a configuration similar to the one for the speckle sieve, the memory effect can be used to exert a perpendicular force to the flow selectively on the larger particles, so that each kind of particle gets guided into a different channel.

## Discussion

Using similar configurations, which are routinely used in microfluiduics, it is possible to separate particles on the basis of various parameters, such as their size or their refractive index, as shown in Supplementary Figures S2 and S3; the resolution of this optical fractionation^17^,^18^,^19^ is only limited by the size of the speckle field, i.e., the longer the speckle field the higher the sensitivity in particle's size or refractive index. These devices can, therefore, be scaled to achieve the high throughput or sensitivity needed in microfluidics (thousands of particles per second) by increasing the flow speed and laser power, as it is also the case for alternative optofluidics devices^17^,^18^,^19^. Moreover, an additional advantage of speckle patterns is that they are also intrinsically widefield, and could be used to sort many particles in parallel in a broader microfluidic chamber, where flow speed is strongly reduced. From the experimental point of view, speckle light fields with such universal statistical properties are straightforwardly generated over large areas using different processes, such as scattering of a laser on a rough surface, multiple scattering in an optically complex medium, or mode-mixing in a multimode fiber^4^. In the Supplementary Note S3, we propose some possible setups that can be implemented at low cost, with little alignment and few optical components based on these processes of generation of a speckle field. Here, we have mainly considered possible experimental implementations where the Brownian particles are moving in a quasi 2-dimensional speckle pattern. This is particularly true for experiments that are designed satisfying one of the following conditions: the speckle field is generated with a low numerical aperture so that the average longitudinal extension of the speckle grain extends over the depth of the microfluidic channel; optical scattering forces push the particles in the direction of propagation of light, so that in the presence of a boundary, such as the glass or PDMS surface of a microchannel, they effectively confine the particles in a quasi two dimensional space^34^; or the motion of the particles is confined to a quasi 2-dimensional volume far from any physical boundary, for example, by employing acoustic standing waves^35^. In more complex settings, our results can be readily extended to take into account a 3-dimensional description of the speckle field^24^. Particular care should be paid to extent the speckle memory effect to a 3-dimensional case: while the standard memory effect achieves a lateral translation of the speckle field by adding a linear phase gradient at the input of a complex medium^21^,^22^, a longitudinal translation of the speckle field can be obtained by adding a quadratic phase gradient at the input^36^. Therefore a combination of these two effects allows one to translate the results of our work to a 3-dimensional situation.

In conclusion, we have developed a theoretical framework that allows one to convert the randomness of a speckle light field into a tunable tool to influence the motion of a Brownian particle. In particular, we performed numerical experiments to show the applicability of this concept to the tunable control of anomalous diffusion and to perform standard optical manipulation tasks, such as sorting and guiding. While some of the tasks we propose in the manuscript can be achieved using optical traps or lattices in periodic arrangements^13^,^14^,^15^,^16^,^17^,^18^,^19^, the alternative use of random potentials offers some additional advantages, such as intrinsic robustness to noise and aberrations from the optics and the environment. In fact, the use of random optical potentials over periodic ones has the advantage of requiring very simple optical setups as well as a very low degree of control over the experimental environment, thus offering the possibility of performing optical manipulation through scattering media, such as diffusers and biological tissue, where light propagation naturally leads to the formation of speckle patterns, without recurring to wavefront shaping^11^.

## Methods

### Simulation of Brownian motion in a speckle pattern

The motion of a Brownian particle of radius *R* in a generic force field can be modeled with the following Langevin equation^37^: 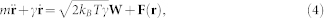
where 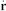, *m* and *γ* = 6*πηR* are respectively the particle's velocity, mass and friction coefficient, *η* the viscosity of the surrounding medium, **W** a white noise vector, *k_B_* the Boltzmann constant and *T* the temperature of the system. For a Rayleigh particle, **F**(**r**) = *κ*∇*I*(**r**) where 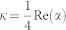 and *α* is the particle polarizability, which depends on the particle's volume, shape and composition^38^. For a spherical particle with radius *R* and refractive index *n_p_* immersed in a liquid with refractive index *n_m_*, 
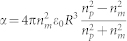
. Inserting the full expression of the force and neglecting inertial effects in the motion of the particle^29^, Equation (4) simplifies as: 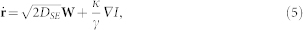
where 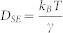 is the Stokes-Einstein's diffusion coefficient of the Brownian particle. Simulations of Brownian motion in the field of forces generated by a speckle pattern were therefore obtained by numerically solving Equation (5)^39^. In all simulations, the particles are polystyrene beads (*n_p_* = 1.59) in water (*n_m_* = 1.33, *η* = 0.001 Ns/m^2^, *T* = 300 K). Due to the low scattering cross-section of the particles and their low concentration, we consider that the force field of the speckle pattern is not influenced by the particle. Even though we used a 2-dimensional model to simulate Brownian dynamics, our results can be readily extended to a 3-dimensional situation by considering a fully 3-dimensional description of the speckle pattern^24^,^39^. In the presence of a hard boundary, an effective diffusion constant and an effective friction coefficient must be included in the model^40^, while for the case of low density suspensions of Brownian particles multiple reflections are very small, and can be safely neglected. In the presence of flow, we assumed laminar flow because of the low Reynolds numbers associated to microfluidc channels^29^,^40^. Every particle trajectory used in the data presented from Figure 1 to 3 was calculated over a different realization of speckle field. Moreover, the initial position for the trajectory was randomly chosen within the speckle field. In Figure 4, instead, we used the same speckle field and initial position for every simulated particle.

### Mean square displacement calculation

The calculation of the MSD is performed according to 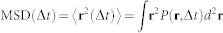, where *P*(**r**,Δ*t*) is the probability density function of finding a particle at position **r** at time Δ*t*^1^. In practice, each MSD curve was obtained by averaging over 500 different particle trajectories that were simulated over 100 s after waiting enough time for the particles to thermalize in the random optical potential given by the speckle pattern.

## Author Contributions

G.V., G.V. and S.G. contributed to the original idea. G.V. and G.V. developed the theoretical model. G.V. (Giorgio Volpe) performed the numerical simulations. All authors discussed the results and contributed to the redaction of the manuscript.

## Supplementary Material

Supplementary Information Supplementary Information

Supplementary Information Supplementary Video S1

Supplementary Information Supplementary Video S2
