# Supplementary material for: Brownian Motion in a Speckle Light Field: Tunable Anomalous Diffusion and Selective Optical Manipulation
Source: Sci Rep. 2014 Feb 5;4:3936. doi: 10.1038/srep03936 (PMC3913929; doi:10.1038/srep03936)
Supplement: Supplementary Information [file srep03936-s1.pdf]

Supplementary Information

Brownian Motion in a Speckle Light Field: Tunable Anomalous Diffusion and Selective Optical Manipulation

Giorgio Volpe[1*], Giovanni Volpe[2] & Sylvain Gigan[1]

1. Institut Langevin, UMR7587 of CNRS and ESPCI ParisTech, 1 rue Jussieu, 75005 Paris, France

2. Physics Department, Bilkent University, Cankaya, 06800 Ankara, Turkey

**Corresponding author: giorgio.volpe@espci.fr*

Contents:

Supplementary Figure S1 – Optical force calculation

Supplementary Figure S2 – Fractionation by size

Supplementary Figure S3 – Fractionation by refractive index

Supplementary Figure S4 – Speckle intensity distribution

Supplementary Figure S5 – Speckle autocorrelation function

Supplementary Figure S6 – Optical force distribution

Supplementary Figure S7 – Possible experimental setups

Supplementary Note S1 – Speckle generation and properties

Supplementary Note S2 – Optical forces in a speckle field

Supplementary Note S3 – Possible experimental configurations

Supplementary Video S1 – Speckle sieve

Supplementary Video S2 – Speckle sorter

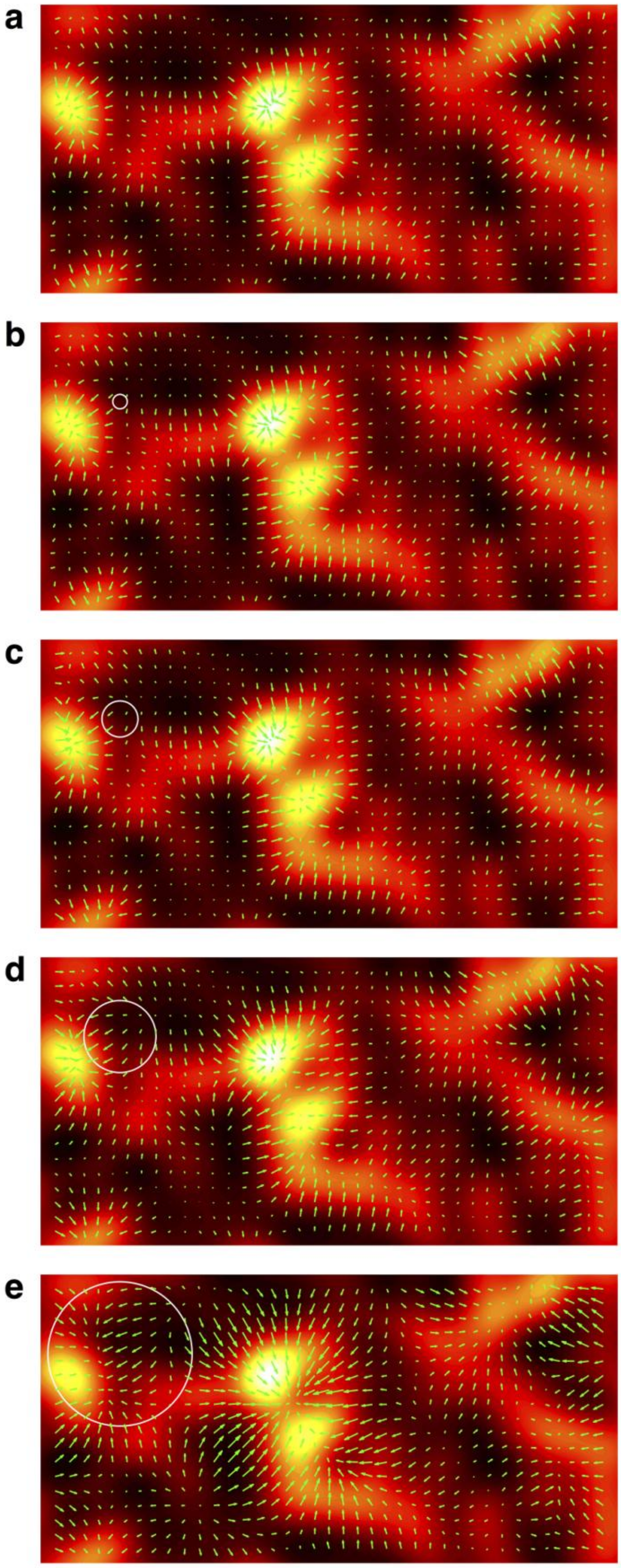

Figure S1: Optical forces in a speckle field for particles of different sizes. Optical forces in the transversal plane (green arrows), calculated using exact electromagnetic

theory[27-28], in a speckle field (background) for polystyrene particles ($n_p = 1.59$) in water ($n_m = 1.33$, η = 0.001 Ns/m^2, T = 300 K) for (**a**) a point dipole where the Rayleigh approximation strictly holds and for particles with increasing radii (**b**) R = 100 nm, (**c**) R = 250 nm, (**d**) R = 500 nm and (**e**) R = 1000 nm. The force field acting on particles whose radius is smaller than the wavelength (**b-d**) is qualitatively the same as the one acting on the point dipole in (**a**). A change in the field happens only for much larger particles: the particle in (**e**) is not attracted to the maxima of intensities in the speckle field but to a minimum of intensity separating two or more speckle grains, as previously observed in periodic patterns[41]. The white circles represent the different particle's sizes. In every plot the length of the arrows representing the force is normalized to its maximum.

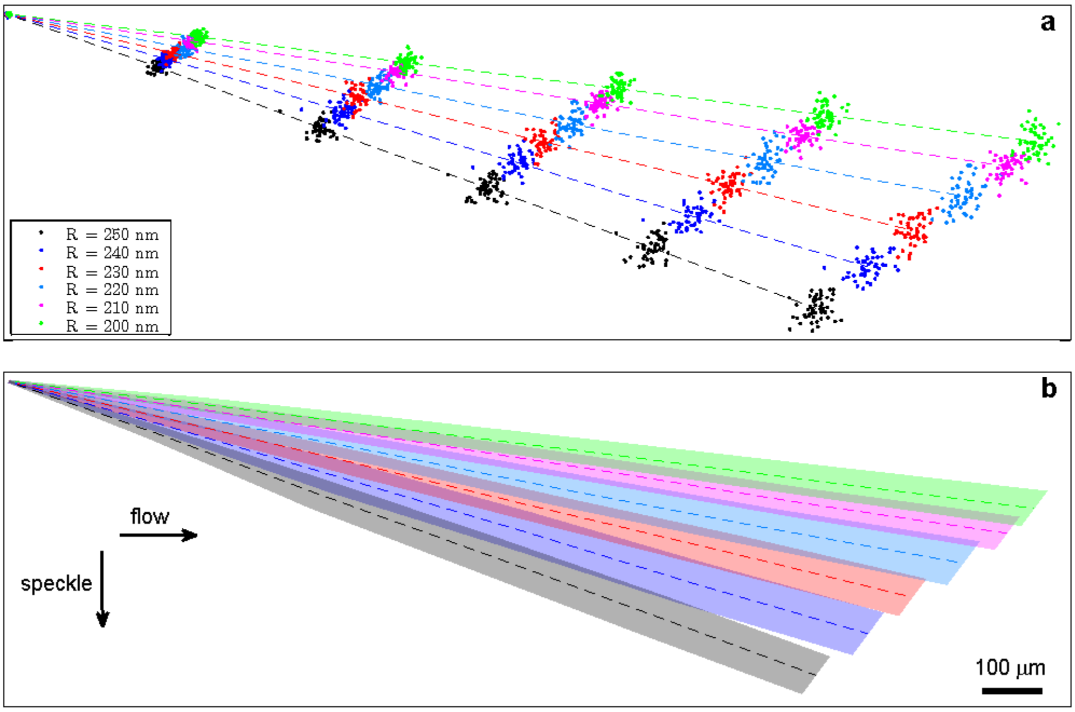

Figure S2: Fractionation by size. (**a**) The color-coded symbols represent the positions of particles of various radii ($n_p = 1.59$, radii varying from 250 nm to 200 nm with 10 nm steps) at different times (shown every 10 seconds) as they propagate along a vertically ratcheting speckle pattern in a channel with a flow from the left (flow speed 34 μm/s). The dashed lines represent the motion of the center of mass for the different groups of particles. (**b**) The shaded areas represent one standard deviation of the particle's spread around the mean value. The black scale bar corresponds to 100 μm. The resolution of the fractionation is only limited by the size of the speckle pattern, i.e., the longer the speckle pattern the higher the sensitivity in particle's size.

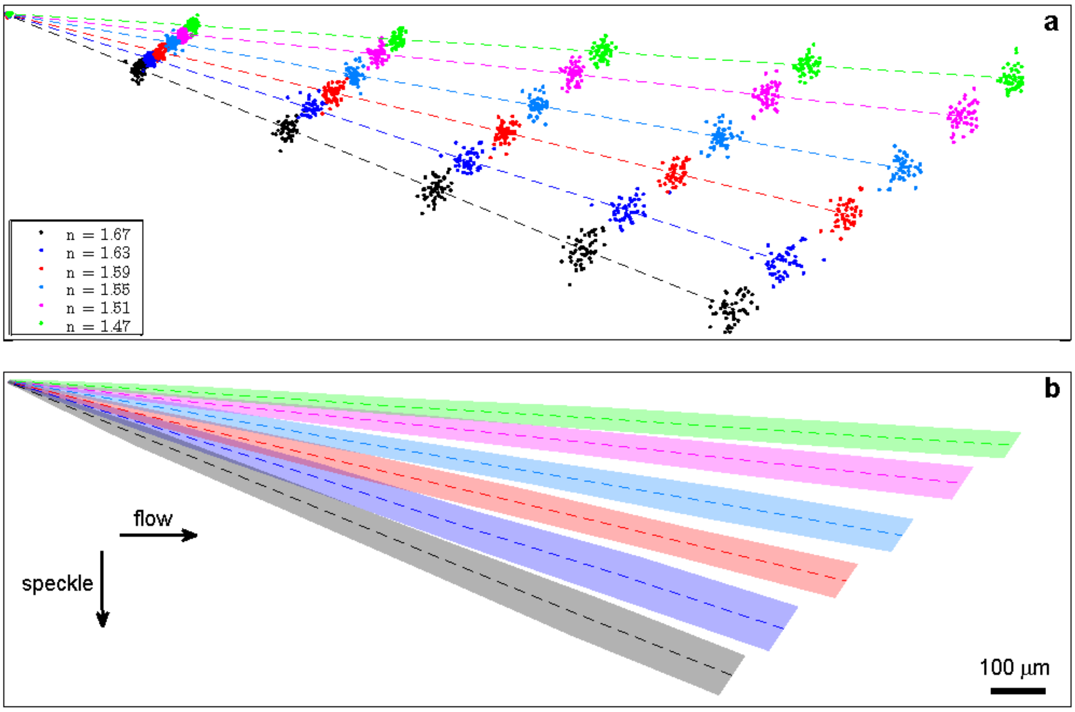

Figure S3: Fractionation by refractive index. (**a**) The color-coded symbols represent the positions of particles of various refractive indexes ($R = 225$ nm, n_p varying from 1.67 to 1.47 with 0.04 steps) at different times (shown every 10 seconds) as they propagate along a vertically ratcheting speckle pattern in a channel with a flow from the left (flow speed 34 μm/s). The dashed lines represent the motion of the center of mass for the different groups of particles. (**b**) The shaded areas represent one standard deviation of the particles' spread around the mean value. The black scale bar corresponds to 100 μm. The resolution of the fractionation is only limited by the size of the speckle pattern, i.e., the longer the speckle pattern the higher the sensitivity in particle's refractive index.

Supplementary Note S1 – Speckle pattern generation and properties

Speckle pattern intensity distribution

A speckle pattern is an interference figure resulting from random scattering of coherent light by a complex medium. The probability density function of the speckle pattern intensity I follows the negative exponential distribution[3,24]:

$$p(I) = \frac{1}{\langle I \rangle} e^{-\frac{I}{\langle I \rangle}}, \qquad (S1)$$

where $\langle I \rangle$ is the average speckle pattern intensity. Figure S4 shows the very good agreement between theoretical and numerical distributions of the speckle pattern intensities used in the simulations.

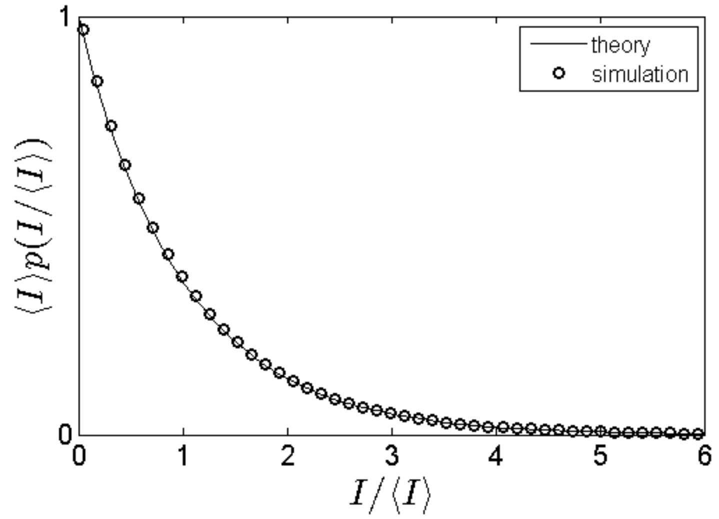

Figure S4: Speckle pattern intensity distribution. Theoretical (solid line) and numerical (circles) probability density function of the speckle pattern intensity I.

Speckle pattern correlation function and its Gaussian approximation

The normalized spatial autocorrelation function of the speckle pattern, which provides a measure of the average speckle grain size, is defined by the diffraction process that generates the speckle pattern itself[3,24]. For a fully developed speckle pattern, in the general case, this autocorrelation function can be approximated by a Gaussian function whose standard deviation depends on the size of the average speckle grain. In what follows, we treat the case of a speckle pattern generated by diffraction through a circular aperture, as used in the simulations. In this case, the autocorrelation function is the Airy disk (Figure S5):

$$C_I(\Delta\mathbf{r}) = \frac{\langle I(\mathbf{r}+\Delta\mathbf{r})I(\mathbf{r})\rangle}{\langle I(\mathbf{r})^2\rangle} = \left|2\frac{J_1(a|\Delta\mathbf{r}|)}{a|\Delta\mathbf{r}|}\right|^2, \qquad (S2)$$

where $I(\mathbf{r})$ is the speckle pattern intensity as a function of the position $\mathbf{r}, J_1$ is the Bessel function of the first kind and order 1 and $a = \frac{2\pi NA}{\lambda}$, being λ the wavelength of light, and NA the numerical aperture under which the speckle pattern is generated. The distance d between $|\Delta\mathbf{r}| = 0$ and the first minimum of the Airy function ($|\Delta\mathbf{r}| = 0.61\frac{\lambda}{NA}$) defines the average speckle grain size at the plane of observation, where NA for a speckle pattern generated at distance z from an area of diameter D is, around the optical axis if $D \gg z$, $NA = n_m \sin\left[\operatorname{atan}\left(\frac{D}{2z}\right)\right]$, where n_m is the refractive index of the medium where the speckle pattern is observed. In our simulations, $D = 1$ mm, $z = 10$ µm, $n_m = 1.33$, and $\lambda = 1064$ nm, giving an average speckle grain size of 490 nm.

Any Airy function is very well approximated by a Gaussian function of standard deviation $\sigma = 0.21\frac{\lambda}{NA}$ so that[25]

$$C_I(\Delta\mathbf{r}) \approx e^{-\frac{|\Delta\mathbf{r}|^2}{2\sigma^2}}, \qquad \text{(S3)}$$

or, making explicit the dependence on x and y

$$C_I(x, y) \approx e^{-\frac{x^2+y^2}{2\sigma^2}}. \qquad \text{(S4)}$$

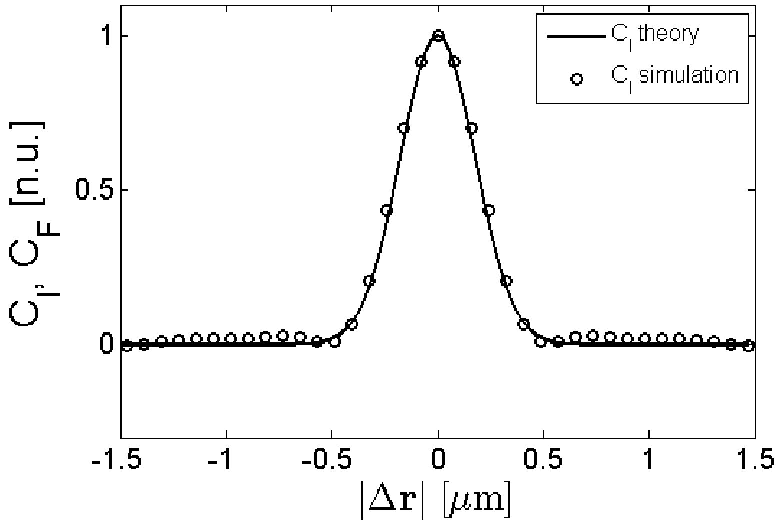

Figure S5: Speckle pattern intensity autocorrelation function. Normalized speckle pattern intensity autocorrelation functions as given by the Gaussian model of Equation (S3) (line), and by the Airy function of the numerically generated speckle pattern (circles).

So far, we have considered the case of 2-dimensional speckle patterns, while a full 3-dimensional description of speckle patterns might be sometimes necessary. As for the case of the 2-dimensional speckle pattern, the statistical properties of the 3-dimensional patterns are also fully defined by the diffraction process that generates them, so that the average size of the 3-dimensional speckle grain can be defined at any point of observation away from the plane where the speckle pattern is generated[24].

Supplementary Note S2 – Optical forces in a speckle pattern: probability density function and correlation function

Force probability density function

In a speckle field, the optical forces exerted on a Brownian particle are proportional to the gradient of the speckle pattern intensity (see Methods). Since the speckle field is known, we can fully derive numerically the associated random force field and calculate its statistical properties. In particular, from the definition of variance, it can be shown numerically that he following property holds:

$$\langle F^2 \rangle = \langle F \rangle^2 + \mathrm{var}(F) = n\langle F \rangle^2, \tag{S5}$$

where F is the absolute value of the force and $n \approx 1.618$. This property will be useful to derive Equation (S10). In Figure S6, we plot the probability density function of the force for different particle' radii, and, as a guide for the eyes, we fit it to the following empirical function:

$$p(F) = A\left(\frac{nF}{\langle F \rangle}\right)^{\frac{1}{n}} e^{-\frac{nF}{\langle F \rangle}}, \tag{S6}$$

where A is a normalization factor.

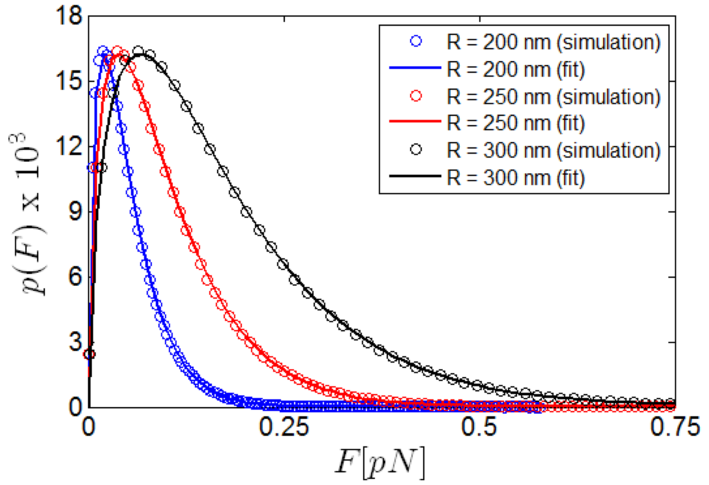

Figure S6: Probability density function of the optical forces in a speckle field. Probability density function of the optical forces (circles) acting on particles of different radii moving on the same speckle field, and fitting to Equation (S6) (lines). The average forces increases with the particle radius, $R = 200$ nm (blue circles and line), $R = 250$ nm (red circles and line) and $R = 300$ nm (black circles and line).

Force correlation function

The aim is to calculate the force correlation function from the intensity correlation function given in Equation (1).

The correlation of the force can be expressed as

$$\begin{aligned} C_F(\Delta\mathbf{r}) &= \langle \mathbf{F}(\mathbf{r}+\Delta\mathbf{r}) \cdot \mathbf{F}(\mathbf{r}) \rangle \\ &= \langle F_x(\mathbf{r}+\Delta\mathbf{r}) \cdot F_x(\mathbf{r}) \rangle + \langle F_y(\mathbf{r}+\Delta\mathbf{r}) \cdot F_y(\mathbf{r}) \rangle \\ &= C_{F_x}(\Delta\mathbf{r}) + C_{F_y}(\Delta\mathbf{r}) \end{aligned}$$

where $\cdot$ represent the dot-product, $\mathbf{F}$ the force vector, and F_x and F_y the force components. We can now Fourier-transform $C_{F_x}(\Delta\mathbf{r})$ and $C_{F_y}(\Delta\mathbf{r})$ so that

$$\tilde{C}_{F_x}(\mathbf{f}) = \left|\tilde{F}_x(\mathbf{f})\right|^2 = \kappa^2\left|2\pi i f_x \tilde{I}(\mathbf{f})\right|^2 = -\kappa^2(2\pi i f_x)^2\left|\tilde{I}(\mathbf{f})\right|^2,$$

and

$$\tilde{C}_{F_y}(\mathbf{f}) = \left|\tilde{F}_y(\mathbf{f})\right|^2 = \kappa^2\left|2\pi i f_y \tilde{I}(\mathbf{f})\right|^2 = -\kappa^2\left(2\pi i f_y\right)^2\left|\tilde{I}(\mathbf{f})\right|^2,$$

Using the Wiener-Khinchin theorem, we can now derive the correlation function of the force from the correlation function of the intensity:

$$C_{F_x}(\Delta\mathbf{r}) = -\kappa^2\langle I\rangle^2\frac{\partial^2 C_I(\Delta\mathbf{r})}{\partial x^2} = \frac{\kappa^2}{\sigma^2}\langle I\rangle^2\left(1-\frac{x^2}{\sigma^2}\right)e^{-\frac{|\Delta\mathbf{r}|^2}{2\sigma^2}},$$

and

$$C_{F_y}(\Delta\mathbf{r}) = -\kappa^2\langle I\rangle^2\frac{\partial^2 C_I(\Delta\mathbf{r})}{\partial y^2} = \frac{\kappa^2}{\sigma^2}\langle I\rangle^2\left(1-\frac{y^2}{\sigma^2}\right)e^{-\frac{|\Delta\mathbf{r}|^2}{2\sigma^2}},$$

so that

$$C_F(\Delta\mathbf{r}) = \frac{\kappa^2}{\sigma^2}\langle I\rangle^2\left(2-\frac{|\Delta\mathbf{r}|^2}{\sigma^2}\right)e^{-\frac{|\Delta\mathbf{r}|^2}{2\sigma^2}}, \qquad \text{(S7)}$$

which is rotationally symmetric. From this function we now can extract the force correlation length

$$L = \sqrt{2}\sigma, \qquad \text{(S8)}$$

which is the value for which the components of the correlation function have the first zero, so where the force change sign on average.

Moreover, we have

$$\langle F^2\rangle = C_F(\mathbf{0}) = 2\frac{\kappa^2}{\sigma^2}\langle I\rangle^2, \qquad \text{(S9)}$$

and since, from Equation (S5), $\langle F^2\rangle = n\langle F\rangle^2$, we finally obtain:

$$\langle F \rangle = \sqrt{\frac{2}{n}}\frac{\kappa}{\sigma}\langle I \rangle \approx \frac{\kappa}{\sigma}\langle I \rangle. \qquad (S10)$$

Supplementary Note S3 – Possible experimental configurations.

From the experimental point of view, speckle light fields with the required statistical properties are routinely generated over large areas using different processes, such as scattering of a laser on a rough surface, multiple scattering in an optically complex medium, or mode-mixing in a multimode fiber[4]. Figure S7 shows two schematics of different possible setups that can be implemented at low cost with little alignment based on two processes to generate a speckle pattern, e.g., light propagation through a multimode optical fiber and light scattering from a complex medium, such as paper, paint, or many biological tissues[4]. The use of a multimode optical fiber, in particular, provides homogeneous speckle fields over controllable areas. Since the random potential of a speckle field results from optical forces, we expect the power requirements per unit of area to be comparable to the ones employed to achieve optical manipulation in periodic potentials[13-19,34,41]. As for other optical trapping techniques, the bigger the particles and the higher the refractive index, the lower is the need for power to achieve a certain level of average force. Therefore, the input power will greatly scale down when increasing the volume of the particles.

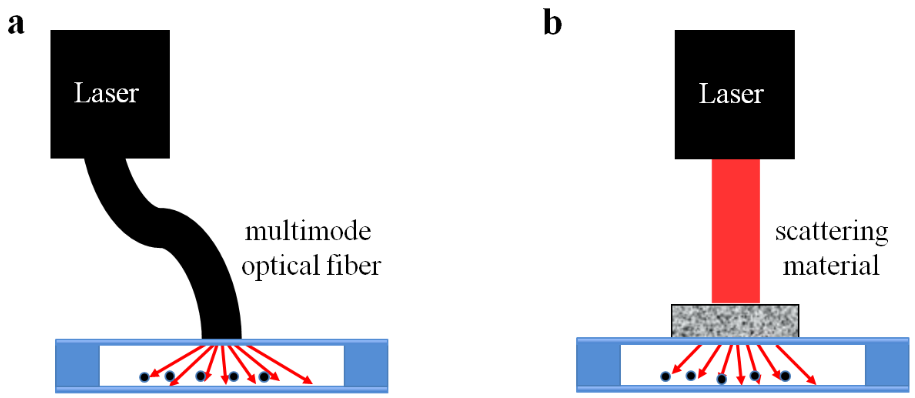

Figure S7: Schematic of possible experimental setups. Two possible setups to perform the proposed experiments in the main text: Brownian particles move within a microfluidic chamber illuminated by a speckle pattern over a broad area. The beam from a laser diode generates the required speckle pattern either (**a**) after propagation in a multimode optical fiber or (**b**) after scattering from an optically complex medium, such as a biological tissue or a thin or thick diffuser.

Finally, the channels we propose in the manuscript are routinely used in microfluidics and optofluidics. Since microfluidic flows are typically laminar and not turbulent (low Reynolds numbers), the flow in these channels can be straightforwardly and accurately predicted and controlled[40].

Supplementary Video S1 – Speckle sieve

Two classes of polystyrene particles with different radii, R = 200 nm (green beads) and R = 250 nm (black beads) are carried from left to right by a flow (42 μm/s). The larger particles are trapped over longer times by a static speckle pattern, here represented by the red disordered area, while the smaller particles tend to escape faster. After 6 s the two classes of particles are completely separated.

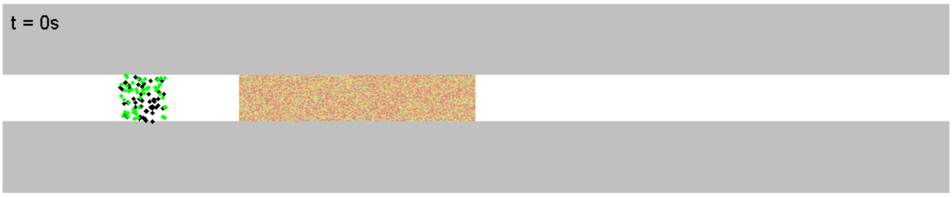

Supplementary Video S2 – Speckle sorter

Two classes of polystyrene particles with different radii, R = 200 nm (green beads) and R = 250 nm (black beads) are carried from left to right by a flow (34 μm/s). The larger particles are pushed down by a speckle pattern shifting over 1 μm with memory effect, here represented by the red disordered area, while the smaller particles keep moving almost on a straight line. After 6 s the two classes of particles are completely sorted.

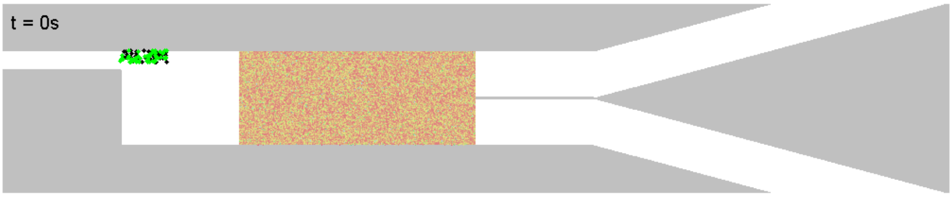

Supplementary References

41. Jákl, P., & Zemánek, P. Particle dynamics in optical lattices, *Proc. SPIE* **7400**, 74000K (2009).
